# Placental cord drainage and its outcomes at third stage of labor: a randomized controlled trial

**DOI:** 10.1186/s12884-022-04877-8

**Published:** 2022-07-18

**Authors:** Nazi Karimi, Ghazaleh Molaee, Najimeh Tarkesh Esfahani, Ali Montazeri

**Affiliations:** 1 Department of Midwifery, Isfahan (Khorasgan) Branch, Islamic Azad University, Isfahan, Iran; 2 Faculty of Humanity Sciences, Payame Noor University, Tehran, Iran; 3 Community Health Research Center, Isfahan (Khorasgan) Branch, Islamic Azad University, Isfahan, Iran; 4 Population Health Research Group, Health Metrics Research Center, Iranian Institute for Health Sciences Research, ACECR, Tehran, Iran; 5 Faculty of Humanity Sciences, University of Science and Culture, Tehran, Iran

**Keywords:** Third stage of labor, Placental cord drainage, Postpartum hemorrhage, Retained placenta, Manual removal of placenta

## Abstract

**Background:**

The third stage of labor begins with the baby’s birth and ends with the expulsion of the placenta and embryonic membranes. The prolongation of the third stage of labor, placental retention, subsequent issues such as postpartum hemorrhage, and manual removal of the placenta have adverse outcomes, which eventually affect the positive experience of delivery. The present study aimed to assess the effect of placental cord drainage on the duration of the third stage of labor and to clarify its effects on postpartum hemorrhage, retained placenta, and incidence of manual removal of placenta.

**Methods:**

This study was a parallel-group randomized trial. Four hundred women in the third stage of labor after vaginal delivery were randomized into the drainage (placenta drainage, *n* = 200) and the control groups (no placenta drainage, *n* = 200). In both groups, the third stage of labor was performed with the active method, and the placenta was removed using the Brandt-Andrews maneuver with maternal pushing. The duration of the third stage was compared between the two groups as the primary outcome. Also, the incidence of postpartum hemorrhage, retained placenta, and manual removal of placenta was compared.

**Results:**

In all, 175 women in the drainage group and 165 women in the control group were included in the analysis. The third stage of labor was significantly shorter after placental cord drainage. The mean duration of the third stage was 7.09 ± 1.01 minutes in the drainage group, and it was 10.43 ± 3.20 minutes in the control group (*P* < 0.001). Postpartum hemorrhage, retained placenta, and incidence of manual removal of placenta in the drainage group was significantly less than in the control group.

**Conclusion:**

Placental cord drainage is a simple and non-invasive method of reducing the duration of the third stage of labor. This method does not increase postpartum complications.

**Trial registration:**

IRCT2014041917341N1, retrospectively registered at 15. 10. 2017.

## Background

The third stage of labor begins with the newborn’s birth and ends with the delivery of the placenta and embryonic membranes [1]. Currently, the usual method of managing the third stage of labor is that both sides of the umbilical cord are clamped and cut, then wait for signs of placental separation; in this time, the common practice is to deliver the placenta with the Brandt-Andrews maneuver [2]. The prolongation of the third stage of labor, placental retention, and subsequent issues such as postpartum hemorrhage, manual removal of the placenta, general anesthesia, and blood transfusions have some adverse outcomes, which eventually might affect the positive experience of a delivery [3, 4]. Accordingly, researchers have been trying to manage the third stage of labor and to recommend the most effective and least risky management method. Based on several suggestions, placental drainage is one of the methods proposed to facilitate placental expulsion. In the drainage method, the clamp at the mother’s side is opened, and the blood of the placenta and the umbilical cord is drained. The drainage of the blood reduces the volume of the placenta, allows the uterus to contract and return to its original state faster, and may reduce the length of the third stage [2].

Few studies have been published about placental drainage. An early randomized study using placenta drainage as a method of placental delivery reported that it significantly reduced the duration of the third stage of labor in vaginal deliveries [5]. Later on, a study confirmed that placental drainage of fetal blood before spontaneous placental delivery significantly reduced the incidence of fetomaternal transfusion in cesarean section [6]. Also, more recent studies indicated benefits for placenta drainage as a part of active management after the third stage of labor after spontaneous vaginal delivery. For instance, a study showed a significant reduction in postpartum blood loss and the duration of the third stage in normal vaginal birth [7]. In contrast, a recent study reported that placental cord drainage had no effect in reducing duration or blood loss during the third stage of labor [8].

However, a review suggested that further studies are needed to reach a definitive conclusion. The review indicated the most important shortcomings to address were the lack of attention to the similarity of the groups studied and the lack of detailed explanation of the method in which the study was conducted [9]. Therefore, we designed this clinical trial to evaluate the effect of placental drainage on the duration of the third stage of labor and determine the safety of this method in terms of retained placenta, the manual removal of the placenta, and postpartum hemorrhage.

## Methods

### Study design

This parallel randomized study examined two methods of management of the third stage of labor in low-risk pregnant women. The Najafabad Islamic Azad University Branch approved the study (IR..IAU.NAJAFABAD.REC.1396.39). All participants signed informed consent at admission.

### Participants

All pregnant women attending three teaching hospitals for delivery in Isfahan, Iran, in 2017 were approached during one complete calendar year. Of these, women were entered into the study if they satisfied the inclusion criteria. The inclusion criteria were singleton pregnancy, term pregnancy (gestational age 37 complete weeks confirmed by certain last mensural period-LMP, or early ultrasound), expected to have a spontaneous vaginal delivery, pregnancy with vertex presentation, absence of placental abnormalities, and fetal congenital anomalies by prenatal ultrasound examination, no maternal obstetric or medical complication such as placental abruption, low lying placenta, oligohydramnios and polyhydramnios, chorioamnionitis, history of prolonged labor (baby is not born after approximately 20 hours of regular contractions), instrumental delivery (vacuum, forceps), antepartum hemorrhage (bleeding from or into the genital tract, occurring from 24 weeks of pregnancy and prior to the birth of the baby), eclampsia and preeclampsia. During the labor, the amount of serum and drugs received, and the length of the first and second stages of labor were recorded. The length of the first and second stages was recorded as a period of hospitalization. In the case of prolonged delivery, instrumental delivery, known coagulation disorders in the mother, and receiving analgesic drugs or anesthesia during labor, the woman was excluded from the study. Cases with a thick size placenta (more than 4 cm) also were excluded.

### Procedure

For all women, active management was used for expulsion of the placenta. After delivery of the fetus-20 units of oxytocin were injected into 1000 ml of lactated ringer’s solution and injected at a speed of 20 ml/min (200 mU/min) for a few minutes until the uterus remained firmly contracted and then was reduced to 2 mL/min until the mother was ready for transfer from the recovery suite to the postpartum unit [10]. The next step was clamping and cutting the umbilical cord. To this end, the control group did not receive any other interventions, and we only calculated the time from cutting the umbilical cord to the full delivery of the placenta. However, for the drainage group, after cutting the umbilical cord, the clamp of the umbilical cord was opened and allowed to drain blood until flow ceased. Recording the time was exactly the same as the previous step. After signs of placental separation, which were a sudden gush of blood into the vagina, a globular and firmer fundus, a lengthening of the umbilical cord as the placenta descends into the vagina, and elevation of the uterus into the abdomen [10], the delivery of the placenta was done by Brandt-Andrews maneuver and encouraging mother to push. This is a technique for expelling the placenta from the uterus during the third stage of labor. One hand puts gentle traction on the cord while the other presses the anterior surface of the uterus backward. After the expulsion of the placenta in both groups, a complete and accurate examination of the placenta and membranes was performed. As such, we checked the uterus regarding the retained placental cotyledon or membranes. In the absence of a placenta expulsion within 30 minutes (retained placenta) [11] or there was suspicion for the retained portions of placenta and membranes after spontaneous separation or in the event of hemorrhage and the necessity of the placenta removal before 30 minutes, it was removed manually. For postpartum hemorrhage after delivery of the placenta and the first 2 hours that the mother was in the recovery unit, we looked at the blood flow from the mother’s vaginal area during the massage of the uterus. We marked bleeding in the checklist as mild, moderate, or severe.

### Outcome measures

Outcome measures included recording the length of the third stage of labor, the incidence of placental retention, the manual removal of the placenta, and postpartum hemorrhage. The recording was carried out by a fellow researcher who was blind to the study.

### Sample size

The following formula was used to estimate the sample size.

$$n=\left(\frac{2\Big({z}_{\alpha /2}+{z_{1-\beta }\Big)}^{2}\times {\sigma}^2}{\varepsilon^2}\right)$$

As such, for a study with an 80% power at 5% significance level and considering precision (ε) by a quarter of the standard deviation (σ), we estimated that the study would require at least 198 pregnant women per each group. The value for Z_α/2_ = 1.96 and Z_1_-_β_ = 0.842 (for 5% significance level and power 80%) are 1.96 and 0.842, respectively. However, in practice, we included 200 women per each group.

### Randomization

Randomization was done in the delivery room. During the data collection period, the study coordinator was responsible for introducing women to the midwife on arrival at the delivery room. The randomization was performed by a midwife who was not involved in the study as a member of the research team. She randomly assigned one pre-marked card to each woman to determine the study groups (intervention or control). The sequence was continued until the required sample size was achieved.

### Statistical analysis

Descriptive statistics, including frequencies, means, and standard deviations, were used to describe the demographic and clinical data. In addition, chi-square, independent samples t-test, the Fisher’s exact test, and Mann-Whitney U test (where necessary) were used for comparison. To control for the baby’s weight, an analysis of covariance was used to compare the length of the third stage between the two groups. To compare delivery outcomes chi-square and the Fisher’s exact tests were applied. In all instances, the level of statistical significance was set at *p* < 0.05.

## Results

### Characteristics of participants

**Fig. 1:**
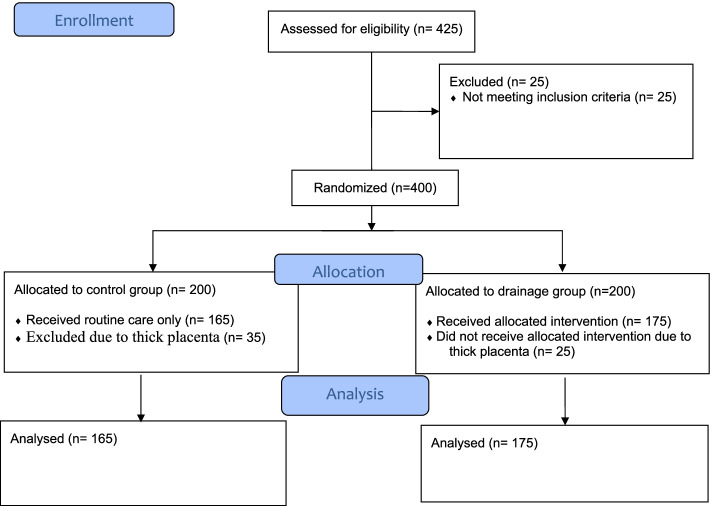
The study flowchart

The study flowchart is depicted in Fig. 1. In all, we have included 175 women in the drainage group and 165 women in the control group (Fig. 1). The mean age of women in the drainage group was 27.45 (SD = 4.57) years, and it was 28.29 (SD = 4.90) for the control group (*P* = 0.103). Similarly, except for neonate’s weight when comparing education, type of pregnancy (wanted or unwanted), medical illness, disease during pregnancy, labor induction, placenta size, gestational age, gravid, and the average stay in the maternity hospital and gravid, no significant differences were observed between the two groups. The detailed results are presented in Table 1.

**Table 1:** The Characteristics of study samples

	Drainage group (***n*** = 175)	Control group (***n*** = 165)	***P*** value
	No. (%)	No. (%)	
**Age** (Mean, SD)	27.4 ± 4.6	28.3 ± 4.9	0.103^a^
**Education**			0.345^b^
Primary	79 (45.1)	75 (45.5)	
Secondary	34 (19.4)	23 (13.9)	
Higher	62 (35.4)	67(40.6)	
**Chronic illness**			0.783^b^
Yes	32 (18.3)	33 (20.0)	
No	143 (81.7)	132 (80.0)	
**Disease during, pregnancy** (e.g. induced hypertension, gestational diabetes)			0.301^b^
Yes	18 (10.3)	23 (13.9)	
No	157 (89.7)	142 (86.1)	
**Labor induction**			0.167^b^
Yes	104 (59.4)	110 (66.7)	
No	71 (40.6)	55 (33.3)	
Placental size*			0.485^c^
Thin (less than 2 cm)	0 (0.0)	1(0.6)	
Normal (2.0 to 2.5 cm)	175 (100.0)	164 (99.4)	
Thick (more than 4 cm)	0 (0.0)	0 (0.0)	
**Gestational age (week)** (Mean, SD)	39.3 ± 0.7	39.6 ± 0.7	0.316^a^
**Gravid** (Mean, SD)	1.74 ± 0.9	1.66 ± 0.9	0.335^d^
**Average stay in the maternity hospital** (Mean, SD in hour)	6.2 ± 3.1	5.5 ± 3.3	0.192^a^
**Neonates’ weight** (Mean, SD in gr)	3063.7 ± 252.2	3184.2 ± 199.6	< 0.001^d^

^*^Measured in delivery room and defined as indicated by the American Family Physician [12]

^a^Independent samples t-test

^b^Chi-square test

^c^Fisher’s exact test

^d^Mann-Whitney U test

### Outcomes and estimations

Comparing the mean values for placental expulsion time in the intervention and control groups, a significant difference (07:09.63 ± 01:01.756 vs. 10:43.37 ± 03:20.512 minutes) was observed. In addition, there were significant differences in manual removal of placenta, retained placenta, and hemorrhage in the study groups indicating favorable outcomes for the intervention group. The detailed results are shown in Table 2.

**Table 2:** The delivery outcomes in intervention and control groups

	Drainage group (***n*** = 175)	Control group (***n*** = 165)	***P***
**The length of third stage of labor**			
**M**ean ± SD (in minutes)	07:09.63 ± 01:01.756	10:43.37 ± 03:20.512	< 0.001^a^
Median	07:10.04	11:00.02	
**Manual removal of placenta** (No., %)			0.001^b^
No	174 (99.4)	152 (92.1)	
Yes	1 (0.6)	13 (7.9)	
**Retained placenta** (No., %)			0.003^b^
No	175 (100.0)	157 (95.2)	
Yes	0 (0.0)	8 (4.8)	
**Hemorrhage** (No., %)			< 0.001^b^
No	175(100.0)	146 (88.5)	
Yes	0 (0.0)	19 (11.5)	

^a^Derived from covariance analysis controlling for neonates’ weights

^b^Derived from Fisher’s exact test

## Discussion

The main purpose of this study was to investigate whether placenta drainage leads to a reduction in the length of the third stage of labor. The second aim was to assess the outcomes of the third stage of labor (placental retention, manual removal of placenta, postpartum hemorrhage) when drainage was performed.

The findings showed that placenta drainage significantly decreased the length of the third stage of labor in the experimental group. Similar results were reported elsewhere [9, 13]. For instance, a systematic review found that placental cord drainage reduced the length of the third stage of labor by a mean of 2.85 minutes and concluded that this was a small but significant reduction in the length of the third stage of labor when cord drainage was applied and compared with no cord drainage. The authors argued that the clinical importance of such detected statistically significant declines is open to discussion and the results should be interpreted with caution [9]. The findings also showed that the mean time of placental removal in both groups was higher than that reported in similar studies [14, 15]. This difference might be due to the fact that in our study, women had higher gestational and maternal age.

The manual removal of the placenta was observed in 7.9% of mothers in the control group, and it was only 0.6% in the experimental group. Such findings also were reported by other investigators [16, 17]. However, a study reported that two cases of the manual removal of the placenta, two cases of retained placenta, and one case of postpartum hemorrhage were observed in the control group. At the same time, none of these conditions were seen in the experimental group. There was no statistically significant difference in the amount of the manual removal of the placenta and retained placenta between the two groups. Still there was a significant difference in bleeding, where the bleeding was higher in the control group [14].

Postnatal outcomes with placental drainage and routine methods also showed that in the experimental group bleeding rate was moderate at 88.5%, while it was moderate at 11.5% in the control group. Placental retention was seen in 4.8% of the mothers in the control group but not in the experimental group. A study found that there was no significant difference in the rate of uterine bleeding and uterine atony, which may be attributed to the small sample size (50 women in each group) [2]. A study with a sample of 200 pregnant women reported that there were no cases of placental retention in the two groups. Postpartum hemorrhage was 3% in the drainage group and 10% in the control group [18]. Similarly, a randomized trial with a sample of 485 women who underwent vaginal delivery in Turkey reported that active management of the third stage of labor with the cord drainage significantly reduced postpartum hemorrhage and the duration of the third stage [19].

A meta-analysis of nine randomized controlled trials comparing placental cord drainage with no cord drainage in the third stage of labor during vaginal delivery showed that compared with clamping the umbilical cord, umbilical cord drainage during the third stage of labor shortened the third-stage duration by 2.28 minutes, but did not reduce the amount of blood loss. The analysis also showed that for women with normal vaginal deliveries, the occurrence of postpartum hemorrhage was reduced by 3%. The authors concluded that placental cord drainage is a simple and non-invasive procedure that should be considered after delayed cord clamping [20].

Finally, one should not ignore the latest WHO recommendations for delayed cord clamping (DCC) of some duration for all babies [21]. As such, it is argued that DCC has many advantages that support this recommendation. For instance, a recent study suggests that DDC beyond 3 min in vaginal deliveries was unrelated to adverse outcomes in babies and showed smaller postpartum blood loss in mothers [22].

Although the current study benefited from a relatively good sample size, it seems that a study with a larger sample size might lead to more precision in findings. The most significant limitation of the current study was the lack of quantifiable data regarding blood loss. Furthermore, adding other outcomes such as psychological outcomes or quality of life measures might add to the value of the findings.

## Conclusion

Compared to the routine hospital procedure, the length of the third stage of labor was shorter when placental drainage was performed. Placental drainage not only did not result in any unsafe outcomes but also reduced the probability of a number of complications such as placental retention, the manual removal of the placenta, and postpartum hemorrhage.

## Data Availability

The dataset used and analyzed during the current study are available from the corresponding authors upon making official request from the Islamic Azad University of Isfahan (Khorasgan Branch).
